# Deep learning from “passive feeding” to “selective eating” of real-world data

**DOI:** 10.1038/s41746-020-00350-y

**Published:** 2020-10-30

**Authors:** Zhongwen Li, Chong Guo, Danyao Nie, Duoru Lin, Yi Zhu, Chuan Chen, Lanqin Zhao, Xiaohang Wu, Meimei Dongye, Fabao Xu, Chenjin Jin, Ping Zhang, Yu Han, Pisong Yan, Haotian Lin

**Affiliations:** 1 State Key Laboratory of Ophthalmology, Zhongshan Ophthalmic Centre, Sun Yat-sen University, 510060 Guangzhou, China; 2 Shenzhen Eye Hospital, Shenzhen Key Laboratory of Ophthalmology, Affiliated Shenzhen Eye Hospital of Jinan University, 518001 Shenzhen, China; 3 Department of Molecular and Cellular Pharmacology, University of Miami Miller School of Medicine, Miami, FL 33136 USA; 4 Sylvester Comprehensive Cancer Centre, University of Miami Miller School of Medicine, Miami, FL 33136 USA; 5 Xudong Ophthalmic Hospital, 015000 Inner Mongolia, China; 6 EYE and ENT Hospital of Fudan University, 200031 Shanghai, China; 7 Centre for Precision Medicine, Sun Yat-sen University, 510060 Guangzhou, China

**Keywords:** Translational research, Medical imaging

## Abstract

Artificial intelligence (AI) based on deep learning has shown excellent diagnostic performance in detecting various diseases with good-quality clinical images. Recently, AI diagnostic systems developed from ultra-widefield fundus (UWF) images have become popular standard-of-care tools in screening for ocular fundus diseases. However, in real-world settings, these systems must base their diagnoses on images with uncontrolled quality (“passive feeding”), leading to uncertainty about their performance. Here, using 40,562 UWF images, we develop a deep learning–based image filtering system (DLIFS) for detecting and filtering out poor-quality images in an automated fashion such that only good-quality images are transferred to the subsequent AI diagnostic system (“selective eating”). In three independent datasets from different clinical institutions, the DLIFS performed well with sensitivities of 96.9%, 95.6% and 96.6%, and specificities of 96.6%, 97.9% and 98.8%, respectively. Furthermore, we show that the application of our DLIFS significantly improves the performance of established AI diagnostic systems in real-world settings. Our work demonstrates that “selective eating” of real-world data is necessary and needs to be considered in the development of image-based AI systems.

## Introduction

Artificial intelligence (AI) platforms provide substantial opportunities to improve population health due to their high efficiencies in disease detection and diagnosis^1–5^. Deep learning, a subset of machine learning based on artificial neural networks, has the ability to self-learn from image features and exhibits remarkable performance in classification tasks^4–12^. Relevant successful applications of deep learning based on different types of images have been reported in health care, such as the detection of diabetic retinopathy in retinal images, with an area under the receiver operating characteristic curve (AUC) of 0.99, and classification of skin cancer in clinical images, with an AUC over 0.91^5,6^.

Currently, in ophthalmology, as ultra-widefield fundus (UWF) imaging becomes a standard-of-care imaging modality for many ocular fundus diseases and a popular tool in screening and telemedicine due to a larger retina area coverage^13–17^, an increasing number of studies have developed deep learning-based AI diagnostic systems for automated detection of ocular fundus diseases using UWF images^18–28^. To date, all previous UWF image-based AI diagnostic systems have been developed and evaluated using good-quality images alone^18–28^. Although the performances of these systems in detecting ocular fundus diseases are ideal in laboratory settings, their performances in real-world settings are unclear because the systems have to make a diagnosis based on images of varying quality. In real clinical scenarios, many factors can compromise image quality, such as patient noncompliance, operator error, hardware imperfections, and obscured optical media^29,30^. Insufficient image quality will result in the loss of diagnostic information and compromise downstream analysis^31–33^. To address this, in the real-world clinic, it is necessary to filter out poor-quality images to ensure that the subsequent AI diagnostic analyses can be based on good-quality images. However, manual image quality analysis often requires experienced doctors and can be time-consuming and labour-intensive, especially in high-throughput settings (e.g., disease screenings and multicentre studies). Therefore, an automated approach to detect and filter out poor-quality images becomes crucial.

In this study, we aimed to develop a deep learning-based image filtering system (DLIFS) to detect and filter out poor-quality UWF images and assess its performance on three independent real-world datasets from different clinical hospitals. In addition, we investigated whether the DLIFS could enhance the performance of our previously established AI diagnostic systems in detecting lattice degeneration/retinal breaks (LDRB), glaucomatous optic neuropathy (GON), and retinal exudation/drusen (RED) using unselected real-world data.

## Results

### Performance of the DLIFS

**Fig. 1:**
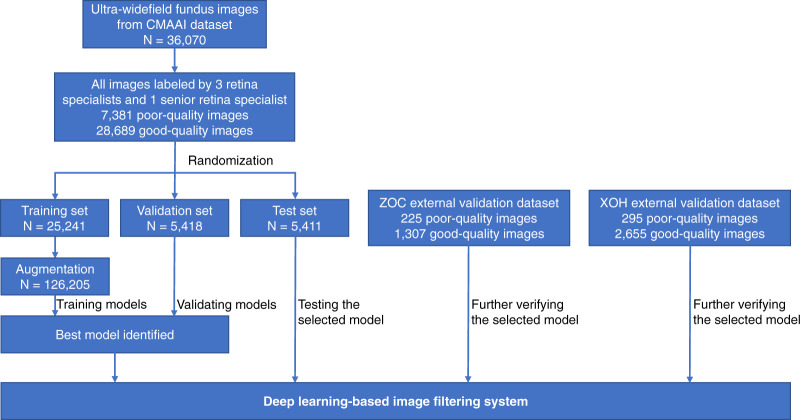
Process of developing and evaluating the deep learning-based image filtering system based on ultra-widefield fundus images. CMAAI Chinese Medical Alliance for Artificial Intelligence, XOH Xudong Ophthalmic Centre, ZOC Zhongshan Ophthalmic Centre.

**Fig. 2:**
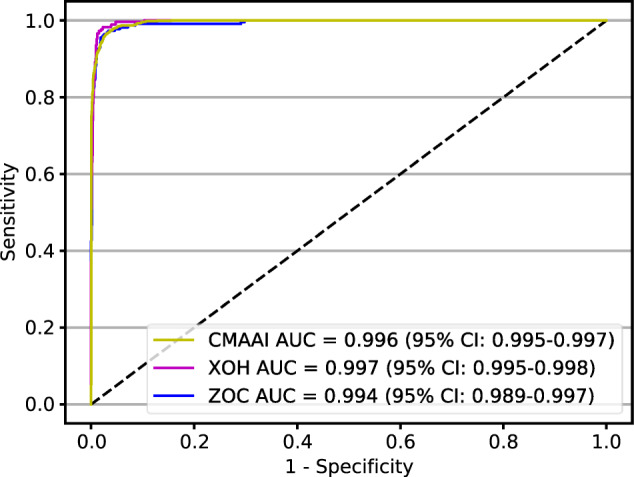
Receiver operating characteristic curves showing the ability of the DLIFS in detecting and filtering out poor-quality ultra-widefield fundus images. AUC area under the receiver operating characteristic curve, CMAAI Chinese Medical Alliance for Artificial Intelligence, DLIFS deep learning-based image filtering system, XOH Xudong Ophthalmic Centre, ZOC Zhongshan Ophthalmic Centre.

In total, 40,562 images from 21,689 individuals aged 3–86 years (mean age of 48.3 years, 44.3% female) were used to develop and evaluate the DLIFS. There were 679 disputed images that were arbitrated by the senior retina specialist, of which, 223 images were assigned to the poor-quality group, and the remaining 456 images were assigned to the good-quality group. Finally, our study included 32661 good-quality images and 7901 poor quality images. Summary information for the datasets from the Chinese Medical Alliance for Artificial Intelligence (CMAAI), Zhongshan Ophthalmic Centre (ZOC), and Xudong Ophthalmic Hospital (XOH) is shown in Fig. 1. The DLIFS for detecting and filtering out poor-quality images achieved AUCs of 0.996 (95% confidence interval [CI]: 0.995–0.997), 0.994 (95% CI: 0.989–0.997), and 0.997 (95% CI: 0.995–0.998) in the CMAAI test set, ZOC set, and XOH set, respectively (Fig. 2). Further information on the model’s performance, including the sensitivity and specificity of each dataset, is displayed in Table 1.

**Table 1:** Performance of the DLIFS in detecting poor-quality images.

	CMAAI test set	Zhongshan ophthalmic centre dataset	Xudong ophthalmic hospital dataset
AUC (95% CI)	0.996 (0.995–0.997)	0.994 (0.989–0.997)	0.997 (0.995–0.998)
Sensitivity (95% CI)	96.9% (96.3–98.3)	95.6% (92.9–98.3)	96.6% (94.5–98.7)
Specificity (95% CI)	96.6% (96.1–97.1)	97.9% (97.1–98.7)	98.8% (98.4–99.2)

*AUC* area under the receiver operating characteristic curve, *CI* confidence interval, *CMAAI* Chinese Medical Alliance for Artificial Intelligence, *DLIFS* deep learning-based image filtering system.

**Fig. 3:**
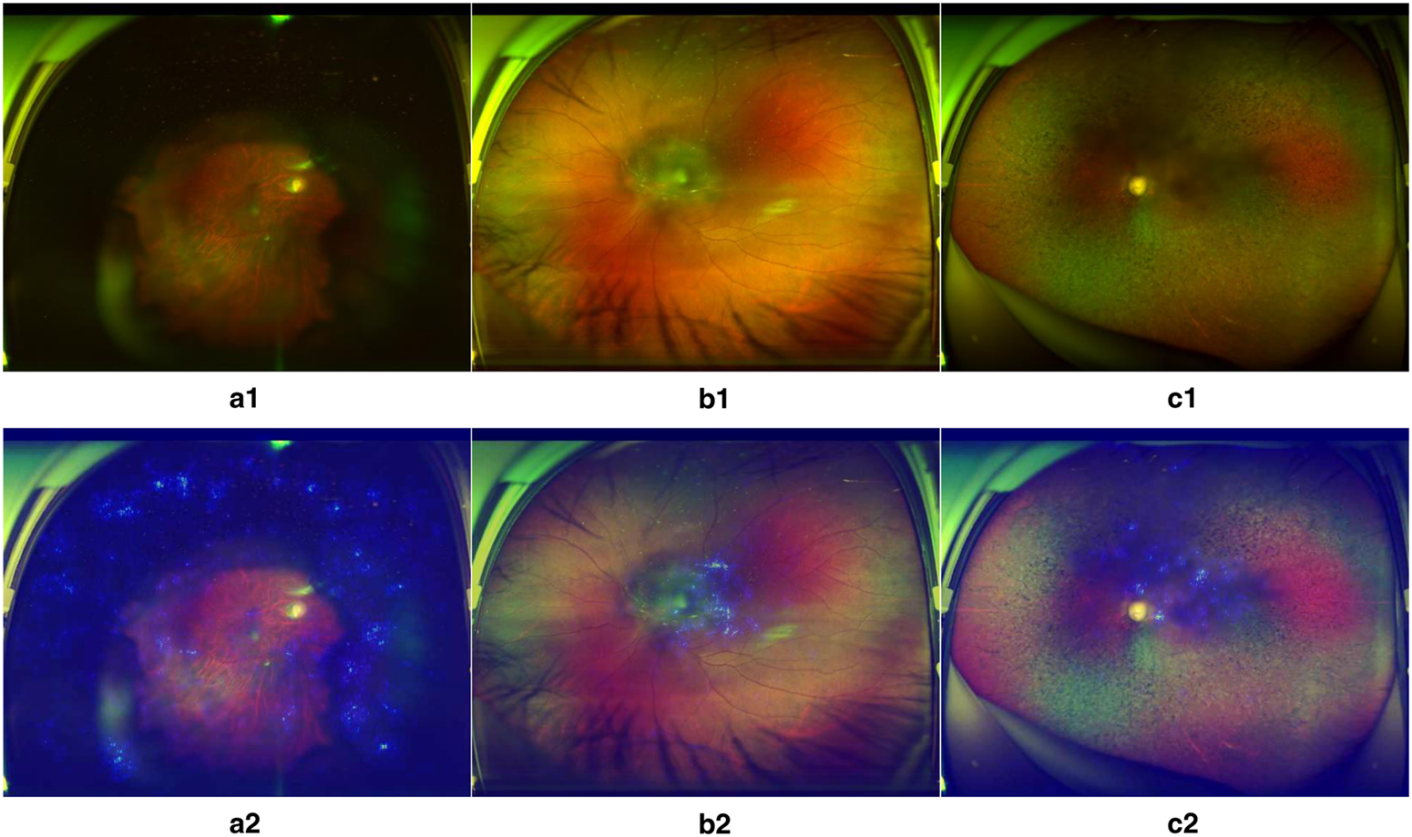
Heatmaps of poor-quality images detected by the DLIFS. Blurred areas shown in original images a1, b1 and c1 correspond to the highlighted regions displayed in heatmaps a2, b2 and c2, respectively. DLIFS, deep learning-based image filtering system.

To visualise how the DLIFS discerned poor-quality images, heatmaps were generated to indicate the poor-quality areas detected by the DLIFS. In the ZOC dataset, the heatmaps effectively highlighted the poor-quality areas regardless of their locations and shapes in all true positive images. Typical examples of heatmaps for poor-quality images are shown in Fig. 3.

### Performances of established AI diagnostic systems with and without the DLIFS

**Fig. 4:**
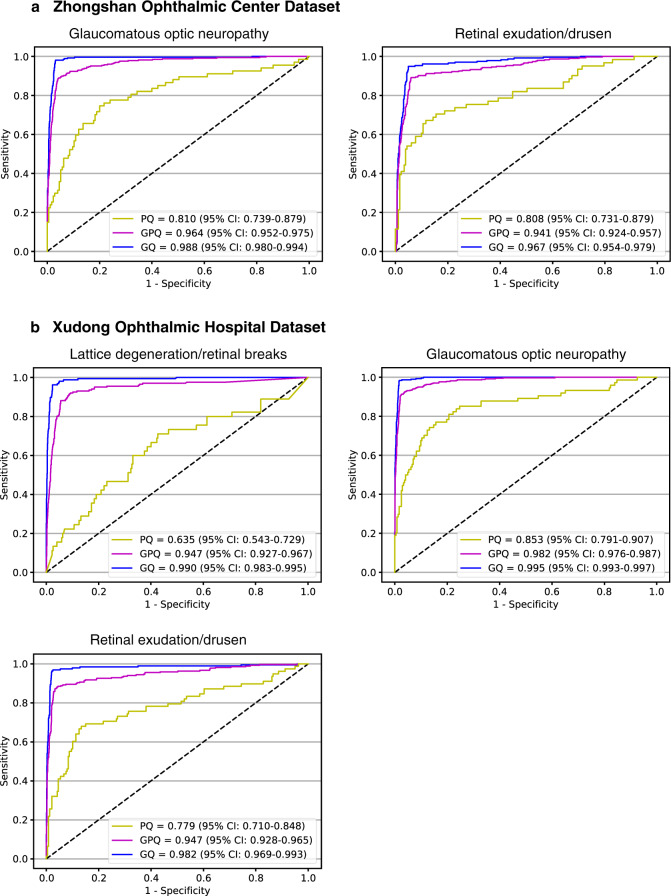
Performances of established AI diagnostic systems in images with different quality levels. Receiver operating characteristic curves of previously established AI diagnostic systems for detecting lattice degeneration/retinal breaks, glaucomatous optic neuropathy, and retinal exudation/drusen in images of only good quality (GQ), only poor quality (PQ) and of both good and poor quality (GPQ), respectively. The images were obtained from the Zhongshan Ophthalmic Centre and Xudong Ophthalmic Hospital datasets. *AI* artificial intelligence.

In the ZOC dataset, the AUCs of the GON system were 0.988 (95% CI: 0.980–0.994), 0.964 (95% CI: 0.952–0.975), and 0.810 (95% CI: 0.739–0.879) in good-quality (with the DLIFS system), mixed-quality (without the DLIFS system), and poor-quality images, respectively (Fig. 4a); the AUCs of the RED system were 0.967 (95% CI: 0.954–0.979), 0.941 (95% CI: 0.924–0.957) and 0.808 (95% CI: 0.731–0.879) in good-quality, mixed-quality and poor-quality images, respectively (Fig. 4A). In the XOH dataset, the AUCs of the LDRB system were 0.990 (95% CI: 0.983–0.995), 0.947 (95% CI: 0.927–0.967) and 0.635 (95% CI: 0.543–0.729) in good-quality, mixed-quality and poor-quality images, respectively (Fig. 4b); the AUCs of the GON system were 0.995 (95% CI: 0.993–0.997), 0.982 (95% CI: 0.976–0.987), and 0.853 (95% CI: 0.791–0.907) in good-quality, mixed-quality and poor-quality images, respectively (Fig. 4b); and the AUCs of the RED system were 0.982 (95% CI: 0.969–0.993), 0.947 (95% CI: 0.928–0.965) and 0.779 (95% CI: 0.710–0.848) in good-quality, mixed-quality and poor-quality images, respectively (Fig. 4b). Details regarding the performance of these systems in the ZOC and XOH datasets are listed in Table 2.

**Table 2:** Performance of previously established AI diagnostic systems in images of varying quality.

	Zhongshan ophthalmic centre dataset				Xudong ophthalmic hospital dataset					
	GON		RED		LDRB		GON		RED	
	Sensitivity (95% CI)	Specificity (95% CI)	Sensitivity (95% CI)	Specificity (95% CI)	Sensitivity (95% CI)	Specificity (95% CI)	Sensitivity (95% CI)	Specificity (95% CI)	Sensitivity (95% CI)	Specificity (95% CI)
Good and poor quality (without DLIFS)	88.6% (85.1–92.1)	95.7% (94.6–96.8)	89.1% (85.5–92.7)	94.0% (92.7–95.3)	86.6% (81.9–91.3)	94.6% (93.8–95.4)	90.3% (87.3–93.3)	97.9% (97.3–98.5)	85.1% (80.8–89.4)	97.3% (96.7–97.9)
Good quality only (with DLIFS)	98.1% (96.4–98.8)	96.6% (95.5–97.7)	94.9% (92.1–97.7)	95.1% (93.8–96.4)	96.2% (93.2–99.2)	97.6% (97.0–98.2)	98.0% (96.4–99.6)	98.5% (98.0–99.0)	95.8% (92.9–98.7)	98.0% (97.4–98.6)
Poor quality only	52.2% (40.2–64.2)	90.3% (85.9–94.7)	67.2% (55.4–79.0)	87.9% (83.1–92.7)	53.3% (38.7–67.9)	67.3% (61.7–72.9)	58.1% (46.9–69.3)	92.6% (89.3–95.9)	59.0% (48.1–69.9)	90.0% (86.2–93.8)

*AI* artificial intelligence, *DLIFS* deep learning-based image filtering system, *GON* glaucomatous optic neuropathy, *RED* retinal exudation/drusen, *LDRB* lattice degeneration/retinal breaks.

After initially applying the DLIFS to detect and filter out the poor-quality images, the sensitivities of the LDRB, GON and RED systems in the two external datasets were increased, and the specificities were comparable to those without applying the DLIFS. Both the sensitivities and specificities of the LDRB, GON and RED systems in good-quality images were better than those in poor-quality images. The detailed results are described in Table 2.

### Differences in disease distribution in poor-quality and good-quality images

**Fig. 5:**
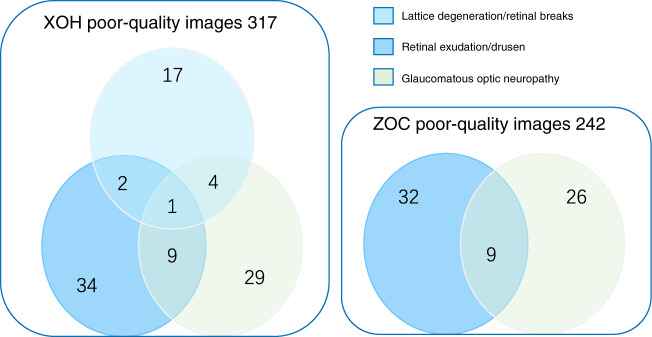
Overlapping ocular fundus diseases in poor-quality images of the XOH and ZOC datasets. The numbers shown in the figure indicate the number of images.

The proportions of GON, RED and LDRB in the poor-quality and good-quality images from the external validation datasets are shown in Table 3. In the ZOC dataset, the proportions of GON and RED that needed to be referred in poor-quality images were significantly higher than those in good-quality images (*P* < 0.05). In the XOH dataset, the proportions of LDRB, GON and RED that needed to be referred in poor-quality images were also significantly higher than those in good-quality images (*P* < 0.05). In total, the established AI diagnostic system indicated that 27.7% (67/242) of the poor-quality images from the ZOC dataset and 30.3% (96/317) of the poor-quality images from the XOH dataset required further clinical investigation. The overlaps among these poor-quality images of LDRB, GON and RED in the ZOC and XOH datasets are described in Fig. 5.

**Table 3:** Proportions of glaucomatous optic neuropathy, retinal exudation/drusen, and lattice degeneration/retinal breaks in the good-quality and poor-quality images.

	ZOC dataset		XOH dataset		
	GON	RED	LDRB	GON	RED
Good quality	258/1290 (20.0%)	233/1290 (18.1%)	156/2643 (5.90%)	306/2643 (8.40%)	190/2643 (7.2%)
Poor quality	67/242 (27.6%)	61/242 (25.2%)	45/317 (14.2%)	74/317 (23.3%)	78/317 (24.6%)
*P* value^*^	0.009	0.01	<0.001	<0.001	<0.001

*ZOC* Zhongshan Ophthalmic Centre, *XOH* Xudong Ophthalmic Centre, *GON* glaucomatous optic neuropathy, *RED* retinal exudation/drusen, *LDRB* lattice degeneration/retinal breaks.

^*^P-values were calculated between the good-quality and poor-quality images using the two-proportion Z-Test.

## Discussion

In this study, after evaluating UWF images from multiple institutions, our DLIFS achieved high sensitivity and specificity in detecting and filtering out poor-quality images. Moreover, our DLIFS had high generalisability as the AUCs were ideal in all the external validation datasets. When the DLIFS is applied in the clinic, photographers can be immediately notified if a poor-quality image is detected, and the photographer can reimage for better quality. If the image quality does not improve after reimaging, obscured optical media will be suspected, and the DLIFS will automatically suggest that the corresponding patient be referred to an ophthalmologist for further examination. Good-quality images, conversely, will be directly transferred by the DLIFS to the subsequent AI diagnostic systems for ocular fundus disease screening.

Several automated techniques for evaluating fundus image quality have been published. Shao et al.^31^ developed a fundus image quality classifier by the analysis of illumination, naturalness, and structure using three secondary indices. Their model achieved a sensitivity of 94.69% and a specificity of 92.29% in 80 images. Hunter et al.^34^ proposed an image quality assessment approach based on the clarity of vessels within the macula area and contrast between macula fovea area and retina background, which achieved a sensitivity of 100% and a specificity of 93% in 200 fundus images. Zago et al.^33^ assessed image quality using a deep learning method and obtained a sensitivity of 95.65% and a specificity of 98.55% in 216 fundus images. Compared with previous studies, there are several important features of our study. First, all previous studies developed image quality evaluation methods based on traditional fundus images. To the best of our knowledge, there is no automated image filtering system to discern poor-quality images for UWF cameras or UWF image-based AI diagnostic systems. This study has developed the DLIFS for detecting and filtering out poor-quality UWF images such that only good-quality images will be transferred to the subsequent AI diagnostic systems (“selective eating process”). Second, to enhance the performance of the DLIFS, the datasets we used to train and validate the DLIFS were substantially large (40,562 UWF images from 21,689 individuals). Third, our datasets were obtained from multiple clinical settings and thereby were more representative of the real world.

Despite the high accuracy of many deep learning-based models, it is very challenging to interpret their output and decision-making rationales^6,8,35^. In this study, the salient regions that the DLIFS used to detect poor-quality images can be highlighted through heatmaps. This interpretability feature of the DLIFS may promote its application in real-world settings as photographers can understand the location of the blurred regions and how a final classification is made by our deep learning algorithm.

Our study demonstrated that previously established AI diagnostic systems performed better in real clinical settings when poor-quality images were detected and filtered out first by the DLIFS. In addition, those AI diagnostic systems exhibited better performances when dealing with good-quality images than with poor-quality images for both external datasets (ZOC and XOH), indicating that the AI diagnostic systems developed based on good-quality images cannot be readily applied to poor-quality images. However, poor-quality images are inevitable in clinical practice due to various factors, such as a dirty camera lens, head/eye movement, eyelid obstruction, operator error, patient noncompliance and obscured optical media^31,36^. Therefore, we propose that the systems developed using good-quality images for detecting retinal diseases in real-world settings (e.g., LDRB, retinal detachment, and retinitis pigmentosa)^18–28^ need to be integrated with the DLIFS to initially discern and filter out poor-quality images, to ensure their optimum performance.

Our DLIFS was developed based on criteria that can be applied to various ocular fundus diseases at different locations (posterior, peripheral, or the entire retina). Therefore, the DLIFS can be readily integrated with other UWF image-based AI diagnostic systems to minimise the negative impacts of poor-quality images. Moreover, cases with poor-quality images detected by the DLIFS will need further evaluation by ophthalmologists because poor-quality images are more likely from patients with ocular fundus diseases (Table 3), which can cause poor target fixation during UWF imaging. Previous reports also suggested the referral of cases with poor-quality images to an ophthalmologist for further evaluation^4,6^.

Although our DLIFS is developed based on a large sample of clinical data from multiple centres, there are still some limitations. First, providing referrals for all cases with poor-quality images will increase the burden on a healthcare system as some results are false positives. An approach that can decrease the number of poor-quality images caused by human factors is needed. In addition, the DLIFS is not capable of identifying the causes of the poor-quality images. It would help photographers to adopt a precise solution if they knew what was leading to the poor quality. Further studies are needed to address this challenge.

In conclusion, our study developed a DLIFS that can accurately distinguish poor-quality UWF images from good-quality ones. The DLIFS can be applied to filter out poor-quality images obtained from real-world settings, thereby significantly improving the ability of AI diagnostic systems trained by good-quality images. As all medical photographic equipment can sometimes produce poor-quality images, irrespective of the field, the process for deep learning from “passive feeding” to “selective eating” real-world data is necessary and needs to be considered when developing an image-based AI diagnostic system.

## Methods

### Ultra-widefield fundus image datasets

A total of 36,070 UWF images (19,684 individuals) were collected from the CMAAI, which is a union of medical organisations, computer science research groups and related enterprises in the field of AI with the aim of improving the research and translational applications of AI in medicine. The CMAAI dataset includes 15322 images obtained from Shenzhen Eye Hospital, 7387 images from Huazhong screening program, 4929 images from Eastern Guangdong Eye Study and 8432 images from Southern China Guangming Screening program. These images were from individuals who presented for retinopathy examinations, ophthalmology consultations, or routine ophthalmic health evaluations, and were obtained between June 2016 and September 2019 using an OPTOS nonmydriatic camera (OPTOS Daytona, Dunfermline, UK) and 200-degree fields of view. Participants were examined without mydriasis. All UWF images were deidentified before they were transferred to research investigators. This study was approved by the Institutional Review Board of ZOC (identifier, 2019KYPJ107) and conducted in accordance with the tenets of the Declaration of Helsinki. Informed consent was exempted due to the retrospective nature of the

data collection and the use of deidentified UWF images.

### Image quality criteria

The image quality was defined as “poor” if any of the following criteria was met:

1. More than one-third of the fundus was obscured^4^.
2. Vessels within the macular area could not be identified or over 50% of the macular area was obscured^8^.
3. Vessels within 1-disc diameter of the optic disc margin could not be identified^37^.

Note: The above criteria for a poor-quality image could be used for lesions scattered throughout the retina (e.g., retinal haemorrhage and exudation), situated in the peripheral retina (e.g., lattice degeneration and retinal breaks), or located at the posterior pole area of the retina (e.g., drusen or glaucoma).

**Fig. 6:**
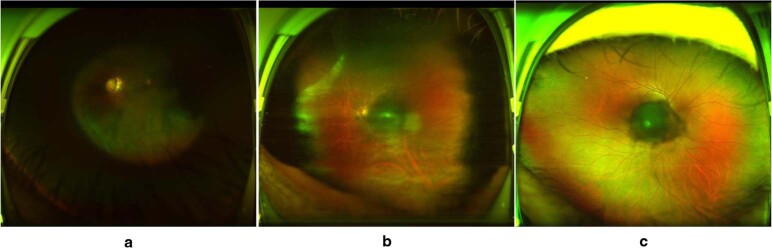
Typical examples of poor-quality ultra-widefield fundus images. **a** Obscured area over one-third of the image. **b** Obscured macular area. **c** Obscured optic disc area.

Examples of poor-quality images are shown in Fig. 6.

If none of the abovementioned criteria was met, the image quality was defined as “good”.

### Image labelling and reference standard

All images were classified into two categories: good quality and poor quality. Three board-certified retina specialists, each with at least five years of clinical experience in UWF image analysis, separately labelled all anonymous images, and they were masked to the DLIFS’s outcomes. For screening out a poor-quality image, they initially assessed whether more than one-third of the image was obscured. If not, they would evaluate whether the vessels within the optic and/ or macular areas could be identified. To ensure the reliability of the image annotation, the reference standard was determined only when a consensus was achieved among all three retina specialists. Any disputed images were presented at a consensus meeting for arbitration by another senior retina specialist with over twenty years of clinical experience to clarify the final classification. The performance of the DLIFS in detecting the poor-quality images was compared to this reference standard.

### Image preprocessing and augmentation

Image standardisation was performed prior to deep feature learning. All images were downsized to 512 × 512 pixels, and the pixel values were normalised to the interval 0–1. Data augmentation was used to increase image heterogeneity of the training dataset and thus reduce the chance of overfitting during the deep learning process. The new samples were obtained through simple transformations of the original images and corresponded to “real-world” acquisition conditions. Random horizontal and vertical flip, random rotations up to 90 degrees around the image centre, and random brightness shift within the range of 0.8 to 1.6 were applied to the images of the training set to increase their size to five times larger than the original size (from 25,241 to 126,205).

### Development and evaluation of the DLIFS

The process for the development and evaluation of the DLIFS is illustrated in Fig. 1. The images from the CMAAI dataset were randomly assigned (7:1.5:1.5) to the training and validation datasets for developing the DLIFS, and the test datasets for evaluating the performance of the DLIFS. No individuals overlapped among these sets. The DLIFS was trained by a state-of-the-art deep convolutional neural network (CNN) architecture, InceptionResNetV2, which combines the architectural characteristics of ResNet and Inception, including skip connection and variable kernel sizes, and results in a more performant architecture than the two predecessors^38^. Our previous study also has demonstrated that the InceptionResNetV2 is the best algorithm for developing an AI system based on UWF images when compared to other three state-of-the-art algorithms (InceptionV3, ResNet50 and VGG16)^19^. Weights pretrained for ImageNet classification were applied to initialise the CNN architectures^39^. The loss function used was binary cross-entropy. The optimiser used was the adaptive momentum optimiser^40^.

The deep learning model was trained up to 180 epochs. In the training process, validation loss was assessed using the validation set after each epoch and applied as a reference for model selection. Early stopping was employed, and when the validation loss did not improve over 60 consecutive epochs, the training process was stopped. The model state with the lowest loss was saved as the final state of the model.

The DLIFS had one input and one output. The input was the UWF images and the output was a standard binary task for determining whether the quality of the input image was poor. Two external datasets were used to further evaluate the performance of the DLIFS. One was derived from the outpatient clinics at ZOC in Guangzhou (southeast China), consisting of 1532 UWF images from 828 individuals, and the other was from the outpatient clinics and health screening centre at Xudong Ophthalmic Hospital (XOH) in Inner Mongolia (northwest China), consisting of 2960 UWF images from 1177 individuals. The reference standard used in these two datasets was the same as the CMAAI dataset.

### Visualisation heatmap

In ZOC datasets, the area of the image that the DLIFS used for classification was highlighted based on the Saliency Map visualisation technique. This technique computes the gradient of the output with respect to the input image to decide which pixels in the input image have more influence on the model’s prediction^41^. The intensity value of the heatmap is a direct indication of the pixels’ impact on the DLIFS’s classification. Using this approach, the heatmap indicates the location on which the decision of the DLIFS is based.

### Evaluation of established AI diagnostic systems with and without the DLIFS

We previously established three AI diagnostic systems based on good-quality UWF images for detecting LDRB, GON, and RED, respectively. The methods used to develop the AI diagnostic systems are as follows: A total of 5915 UWF images (3417 individuals) collected from the CMAAI between November 2016 and February 2019 were used to develop a deep learning system for detecting GON. A total of 22,411 images (13,258 individuals) obtained from CMAAI between June 2016 and June 2019 were used to develop a deep learning system for identifying RED. The distribution of the datasets used to develop these systems is summarised in Supplementary Table 1. Because retinal hard exudation is difficult to distinguish from drusen based on appearance alone and because cases with each of these conditions should be referred, we assigned them to the same group. Methods for developing a deep learning system to detect LDRB were described in our previous study^19^. For GON detection, images were classified into two categories: GON and non-GON. The criteria for diagnosing GON were based on a previous study from Zhongshan Ophthalmic Centre^8^. Poor-quality images for GON were defined when vessels within 1-disc diameter of the optic disc margin could not be identified or over one-third of the photograph was obscured. Poor-quality images were removed before training the deep learning system. For RED identification, the images were assigned to two groups: RED and Non-RED. Poor-quality images for RED were defined when vessels within the macular area could not be identified, when over 50% of the macular area was obscured, or when more than one-third of the photograph was obscured. Poor-quality images were excluded before training the deep learning system. The approach used to develop the AI diagnostic systems was the same as the method used to develop the DLIFS. The performance of these AI diagnostic systems is shown in the Supplementary Fig. 1.

**Fig. 7:**
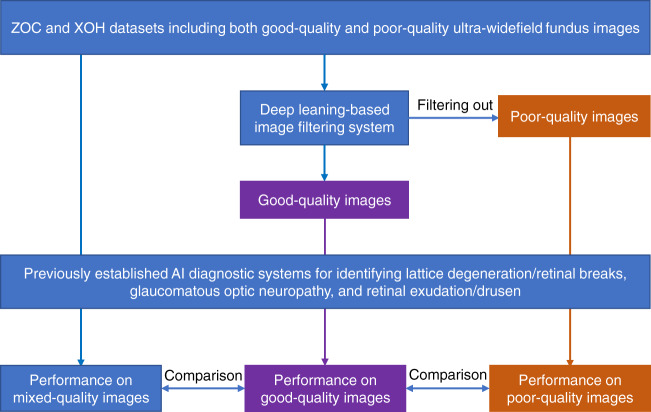
Flowchart evaluating the performance of previously established AI diagnostic systems with good-quality (with DLIFS), mixed-quality (without DLIFS), and poor-quality ultra-widefield images. *AI* artificial intelligence, *DLIFS* deep learning-based image filtering system, *XOH* Xudong Ophthalmic Centre, *ZOC* Zhongshan Ophthalmic Centre.

In the two real-world external datasets (ZOC and XOH) that did not exclude any poor-quality images, the performance of those AI diagnostic systems (LDRB, GON and RED systems) without the DLIFS (images mixed with good and poor quality) was compared to that of the systems with the DLIFS (only good-quality images). The performance of the AI diagnostic systems in only poor-quality images was also evaluated. The detailed research protocol is described in Fig. 7. The actual retinal condition of the poor-quality image cases was obtained from the electronic medical record system. Notably, the ZOC dataset could not be used to evaluate the performance of the LDRB system because this system was trained using part of the ZOC images. There is no overlap between the training data of GON, LBRD, RED model and the XOH dataset, and between the training data of GON, RED and the ZOC dataset in this study.

### Statistical analyses

The deep learning was performed using Keras 2.2.4 with Tensorflow 1.13 as backend. Statistical analyses were conducted using Python 3.7.3 (Wilmington, Delaware, USA). The performance of the DLIFS in detecting poor-quality images was evaluated by calculating the sensitivity, specificity and AUC. The 95% CIs for sensitivity and specificity were calculated with Wilson Score method using a Statsmodels package, and for AUC, using Empirical Bootstrap with 1000 replicates. We plotted a receiver operating characteristic (ROC) curve to show the ability of the DLIFS. The ROC curve was created by plotting the ratio of true positive cases (sensitivity) against the ratio of false-positive cases (1-specificity) using the packages of Scikit-learn and Matplotlib. A larger area under the ROC curve (AUC) implied better performance. Differences of GON, RED and LDRB distribution between poor-quality and good-quality images were calculated using two-proportion Z-Test with the Statsmodels package. All statistical tests were 2-sided with a significance level of 0.05.

### Reporting summary

Further information on experimental design is available in the Nature Research Reporting Summary linked to this article

## Supplementary information

Supplementary Information

Reporting Summary

## Data Availability

The datasets generated and/or analysed during this study are available upon reasonable request from the corresponding author.

## Code availability

The code used in this study can be accessed at GitHub (https://github.com/zocai/uwf-filtering).
