# Supplementary material for: Deep learning from “passive feeding” to “selective eating” of real-world data
Source: NPJ Digit Med. 2020 Oct 30;3:143. doi: 10.1038/s41746-020-00350-y (PMC7603327; doi:10.1038/s41746-020-00350-y)
Supplement: Supplementary file 1 — Supplementary Information [file 41746_2020_350_MOESM1_ESM.pdf]

Supplementary Information

After excluding 652 poor-quality images, 5,263 images were used to develop the system for detecting GON. After excluding 2,520 poor-quality images, 19,891 images were used to develop the system for discerning RED. The distribution of the datasets used to develop the systems is summarized in Supplementary Table 1.

Supplementary Table 1. Data Distribution in the Training, Validation and Test Datasets

	Training Set No. (%)	Validation Set No. (%)	Test Set No. (%)
System for GON detection			
GON	591 (15.9)	125 (16.3)	118 (15.1)
Non-GON	3121 (84.1)	643 (83.7)	665 (84.9)
Total (origin)	3712 (100)	768 (100)	783 (100)
Total (augmentation)*	18560	NA	NA
System for RED detection			
RED	1972 (14.1)	406 (13.7)	415 (14.1)
Non-RED	12013 (85.9)	2548 (86.3)	2537 (85.9)
Total (origin)	13985 (100)	2954 (100)	2952 (100)
Total (augmentation)*	69925	NA	NA

GON, glaucomatous optic neuropathy; RED, retinal exudation/drusen; NA, not applicable;
*Augmentation is approximately five times the original size.

For the classification of GON, the system achieved an AUC of 0.999 with a sensitivity of 97.5% and a specificity of 98.4%. For the detection of RED, the system achieved an AUC of 0.994 with a sensitivity of 94.2% and a specificity of 97.4%. For the identification of LDRB, the system achieved an AUC of 0.999 with a sensitivity of 98.7% and a specificity of 99.2%. More detailed information about the system used for LDRB detection is provided in our previous study[1]. The performance of the systems is shown in Supplementary Figure 1.

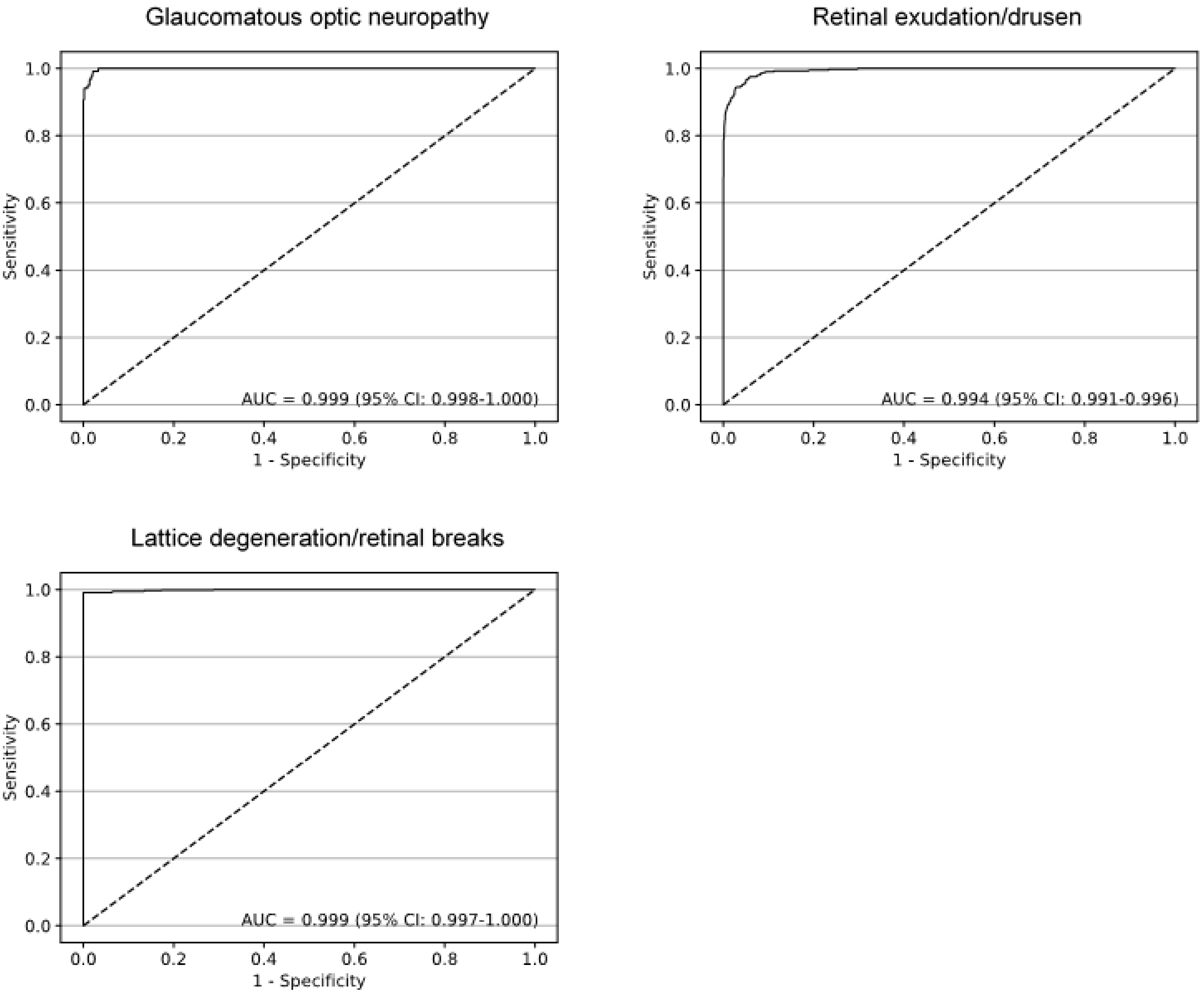

Supplementary Figure 1. Receiver operating characteristic curves of the AI diagnostic systems for detecting glaucomatous optic neuropathy, retinal exudation/drusen and lattice degeneration/retinal breaks based on ultra-widefield fundus images in the test sets.
