# Supplementary material for: Deep learning from “passive feeding” to “selective eating” of real-world data
Source: NPJ Digit Med. 2020 Oct 30;3:143. doi: 10.1038/s41746-020-00350-y (PMC7603327; doi:10.1038/s41746-020-00350-y)
Supplement: Supplementary file 2 — Reporting Summary [file 41746_2020_350_MOESM2_ESM.pdf]

nature research

Corresponding author(s): Haotian Lin

Last updated by author(s): 2020-09-20

Reporting Summary

Nature Research wishes to improve the reproducibility of the work that we publish. This form provides structure for consistency and transparency in reporting. For further information on Nature Research policies, see Authors & Referees and the Editorial Policy Checklist.

Please do not complete any field with "not applicable" or n/a. Refer to the help text for what text to use if an item is not relevant to your study. For final submission: please carefully check your responses for accuracy; you will not be able to make changes later.

Statistics

For all statistical analyses, confirm that the following items are present in the figure legend, table legend, main text, or Methods section.

n/a	Confirmed	
☐	☒	The exact sample size (n) for each experimental group/condition, given as a discrete number and unit of measurement
☐	☒	A statement on whether measurements were taken from distinct samples or whether the same sample was measured repeatedly
☐	☒	The statistical test(s) used AND whether they are one- or two-sided<br>*Only common tests should be described solely by name; describe more complex techniques in the Methods section.*
☒	☐	A description of all covariates tested
☒	☐	A description of any assumptions or corrections, such as tests of normality and adjustment for multiple comparisons
☐	☒	A full description of the statistical parameters including central tendency (e.g. means) or other basic estimates (e.g. regression coefficient) AND variation (e.g. standard deviation) or associated estimates of uncertainty (e.g. confidence intervals)
☐	☒	For null hypothesis testing, the test statistic (e.g. F, t, r) with confidence intervals, effect sizes, degrees of freedom and P value noted<br>*Give P values as exact values whenever suitable.*
☒	☐	For Bayesian analysis, information on the choice of priors and Markov chain Monte Carlo settings
☒	☐	For hierarchical and complex designs, identification of the appropriate level for tests and full reporting of outcomes
☐	☒	Estimates of effect sizes (e.g. Cohen's d, Pearson's r), indicating how they were calculated

Our web collection on statistics for biologists contains articles on many of the points above.

Software and code

Policy information about availability of computer code

Data collection	No software was used.
Data analysis	The performance of the DLIFS in detecting poor-quality images was evaluated by calculating the sensitivity and specificity with 95% confidence intervals (CIs). We plotted a receiver operating characteristic (ROC) curve to show the ability of the DLIFS. The ROC curve was created by plotting the ratio of true positive cases (sensitivity) against the ratio of false-positive cases (1-specificity). All statistical tests were 2-sided with a significance level of 0.05. Statistical analyses were conducted using Python 3.7.3 (Wilmington, Delaware, USA).

For manuscripts utilizing custom algorithms or software that are central to the research but not yet described in published literature, software must be made available to editors/reviewers. We strongly encourage code deposition in a community repository (e.g. GitHub). See the Nature Research guidelines for submitting code & software for further information.

Data

Policy information about availability of data

All manuscripts must include a data availability statement. This statement should provide the following information, where applicable:

- Accession codes, unique identifiers, or web links for publicly available datasets
- A list of figures that have associated raw data
- A description of any restrictions on data availability

The data in this study include the training dataset and the test datasets. Correspondence and requests for data materials should be addressed to HTL (haot.lin@hotmail.com).

Field-specific reporting

Please select the one below that is the best fit for your research. If you are not sure, read the appropriate sections before making your selection.

☒ Life sciences ☐ Behavioural & social sciences ☐ Ecological, evolutionary & environmental science

Life sciences study design

All studies must disclose on these points even when the disclosure is negative.

Sample size	In total, 40,562 images from 21,689 individuals were used to develop and evaluate the DLIFS. The images were obtained between June 2016 and September 2019 using an OPTOS nonmydriatic camera (OPTOS Daytona, Dunfermline, UK) and 200-degree fields of view. All UWF images were de-dentified before they were transferred to research investigators.
Data exclusions	There is no data excluded in this study.
Replication	A state of the art deep CNN architecture, InceptionResNetV2, which mimics the architectural features of two previous CNNs (the Residual Network and the Inception Network), was used to train the DLIFS. Weights pretrained for ImageNet classification were applied to initialize the CNN architectures. The deep learning model was trained up to 180 epochs. In the training process, validation loss was assessed using the validation set after each epoch and applied as a reference for model selection. Early stopping was employed, and when the validation loss did not improve over 60 consecutive epochs, the training process was stopped. The model state with the lowest loss was saved as the final state of the model.
Randomization	To train the DLIFS, the images from the CMAAI dataset were randomly assigned (7:1.5:1.5) to the training and validation datasets for developing the DLIFS, and the test datasets for evaluating the performance of the DLIFS. No individuals overlapped among these sets.
Blinding	Three board-certified retina specialists with at least five years of clinical experience separately labeled all anonymous images, and they were masked to the DLIFS' outcomes.

Reporting for specific materials, systems and methods

We require information from authors about some types of materials, experimental systems and methods used in many studies. Here, indicate whether each material, system or method listed is relevant to your study. If you are not sure if a list item applies to your research, read the appropriate section before selecting a response.

Materials & experimental systems

n/a	Involved in the study
☒	☐ Antibodies
☒	☐ Eukaryotic cell lines
☒	☐ Palaeontology
☒	☐ Animals and other organisms
☐	☒ Human research participants
☐	☒ Clinical data

Methods

n/a	Involved in the study
☒	☐ ChIP-seq
☒	☐ Flow cytometry
☒	☐ MRI-based neuroimaging

Human research participants

Policy information about studies involving human research participants

Population characteristics	In total, 40,562 images from 21,689 individuals aged 3-86 years (mean age 48.3 years, 44.3% female) were used to develop and evaluate the DLIFS.
Recruitment	The images were obtained from CMAAI, ZOC and XOH datasets between June 2016 and September 2019 using an OPTOS nonmydriatic camera (OPTOS Daytona, Dunfermline, UK) and 200-degree fields of view.
Ethics oversight	This study was approved by the Institutional Review Board of Zhongshan Ophthalmic Center (ZOC) and conducted in accordance with the tenets of the Declaration of Helsinki.

Note that full information on the approval of the study protocol must also be provided in the manuscript.
